# Effect of High-Pressure Homogenization on the Properties and Structure of Cold-Induced Chiba Tofu Gel in Soy Protein Isolate

**DOI:** 10.3390/gels10020099

**Published:** 2024-01-26

**Authors:** Li Zheng, Joe M. Regenstein, Zhongjiang Wang

**Affiliations:** 1 College of Food Science, Northeast Agricultural University, Harbin 150030, China; wzjname@neau.edu.cn; 2 Heilongjiang Beidahuang Green Health Food Co., Ltd., Kiamusze 154007, China; 3 Department of Food Science, Cornell University, Ithaca, NY 14853-7201, USA; jmr9@cornell.edu

**Keywords:** soy protein isolate, high-pressure homogenization, structure, gel properties

## Abstract

In the actual production process of soy protein isolate (SPI), most of the homogeneous operating pressure is controlled below 20 MPa due to the consideration of production safety and the limitation of the pressure control capability of homogeneous equipment. In order to improve the functional properties of SPI and adapt it to actual production, the effects of different homogeneous pressures (4, 8, 10, 12, and 14 MPa) on the structure and gel properties of SPI were studied from the perspective of production control. Compared to the control group, the modified SPI improved the hardness, springiness, cohesiveness, chewiness, and water holding capacity (WHC) of the protein gel (*p* < 0.05). Rheological analysis shows that both G′ and G″ increase with increasing frequency, reaching a maximum at 12 MPa. The gel intermolecular force results show that the disulfide bond, hydrophobic interaction, and non-disulfide bond are important molecular forces for gel formation. The particle size distribution uniformity of modified SPI was high, and scanning electron microscopy (SEM) analysis showed that the protein gel with a continuous uniform and dense network structure could be formed by high-pressure homogeneous modification. Overall, high-pressure homogenization technology has the potential to improve SPI gel structure and WHC, and 12 MPa modified SPI gel has the most significant effect.

## 1. Introduction

It is expected that by 2030, China’s supply gap for agricultural products will reach 90 million tons, while that for meat will reach 38.4 million tons. Supply and demand will present a serious imbalance, and a sustainable food supply is facing a huge challenge. Under this background, soy protein food has become a research hot spot [1]. Protein components play an extremely important role in the formation of quality, texture, and flavor in many complex food systems, especially those with protein as the main structure, such as food gels. Soy protein isolate (SPI) is a natural plant protein rich in essential amino acids that is easily digested and absorbed [2]. It is widely used in food processing due to its high protein content and functional properties [3]. In addition, due to the low price of SPI, production costs can be reduced to a certain extent [4,5]. With the improvement of people’s living standards and the change in the concept of a healthy diet, SPI has become popular all over the world with various kinds of vegetarian and low-calorie foods [6]. Chiba tofu, a novel vegetarian food made from traditional tofu products using SPI and starch as the main ingredients, is produced through a relatively simple process [7]. This reduces the processing difficulty and production cost. Chiba tofu is gaining popularity in Asian countries due to its delicious taste and high nutritional content [8]. Currently, the quality of the commercial SPI produced by different manufacturers is unstable due to the differences in its process parameters. There is still a certain gap in the gelatinicity of SPI that needs to be further improved.

Modifying SPI is an effective approach for enhancing its functional characteristics. Some researchers began to actively explore and try to find a simple, efficient, and feasible SPI modification technology. Zhu et al. [9] investigated the effect of MTGase cross-linking on the quality characteristics and potential sensitization of tofu gel. This study showed that the water holding capacity (WHC) and yield of tofu increased significantly after MTGase crosslinking. The sensitization of tofu was also reduced. However, tofu produced using MTGase tends to have a lower gel hardness [10], and the process is often time-consuming [11,12]. Other researchers have explored the effects of chemically modifying SPI. The principle of chemical modification is based on improving the structure, static charge, and hydrophobic group distribution of SPI and then improving its functional properties. The 11S denaturation, dissociation, and elongation of SPI can be induced by acid treatment, but its mode has little influence on 7S. It should be noted that the nutritional value and safety of products obtained through chemical modification require in-depth monitoring and research before they are applied to the actual production of the food system. 

High-pressure homogenization is an effective and safe physical means of modifying the structure and self-assembly properties of proteins. When the high-speed fluid passes through the homogenizing valve, the fluid is restricted by the slit, resulting in high-speed shear force and impact force. The shear and impact forces break, disperse, and homogenize the particles, cells, or colloids in the sample, resulting in particle size reduction, uniform dispersion, and improved stability of the sample. Pressure treatment causes protein denaturation and varying degrees of aggregation or gelation, resulting in changes in textural properties that can extend shelf life [13]. Liu et al. [14] used soybean meal suspensions of different concentrations (15% and 20%) for two or three cycles of high-pressure homogenization (100 and 150 MPa) to study the microstructure of GDL-induced tofu. The results showed that tofu made from 20% soy powder suspension subjected to three cycles at 150 MPa had a denser network with a regular honeycomb structure, and no large particles were found. Chen et al. [15] evaluated the effect of high-pressure homogenization treatment (40–160 MPa) at pH 7.0 on the emulsifying ability of SPI hydrolysates and found that the pancreatic enzymatic hydrolysis of SPI was significantly improved, thus improving the function of the hydrolysates. The study by Fan et al. [16] showed that high-pressure homogenization could reduce the allergenic proteins in soybean flour from large molecules to small molecules, thus reducing anaphylaxis from soybean flour. At present, most of the research on the effect of high-pressure homogenization treatment on SPI modification focuses on improving or enhancing the physical and chemical properties and functional properties of products under high pressure (40–800 MPa). It should be noted that in the actual production process of SPI, due to production safety considerations and the limitation of the pressure control ability of the homogenizing equipment, the homogenizing operating pressure is usually lower than 40 MPa, and most of the homogenizing operating pressure is controlled below 20 MPa [17]. Therefore, from the perspective of production guidance, research on the influence of modification in this pressure range on the structural characterization and functional properties of SPI will have more practical guidance and practical significance. 

In this study, we aimed to improve the gel properties of SPI by using a pressure range closer to actual production (4–14 MPa). Structural changes were studied by SDS-PAGE electrophoresis, Fourier transform infrared spectroscopy (FT-IR), fluorescence spectroscopy, and scanning electron microscopy (SEM). The gel properties of SPI were investigated by texture analysis, water holding capacity (WHC), rheological properties, and gel intermolecular forces. The internal relationship of “modification means—structural change—functional properties” is explained in depth and based more on actual production and easy operation, which provides certain guidance for researchers and manufacturers.

## 2. Results and Discussion

### 2.1. Effect of Homogeneous Modification on Particle Size Distribution

Based on the complexity of the food dispersion system and the difference in particle size and dispersion, the change in gel and stability of the food dispersion system were significantly affected [18]. At present, the laser light scattering technique is widely used in protein and starch dissolution particle size measurement, which can be divided into two kinds: static and dynamic. In this study, the dynamic light scattering technique was used to analyze and test the influence of different high-pressure homogenization treatments on the dissolved particle size of SPI. As shown in Table 1, different degrees of high-pressure homogenization have significant effects on the particle size and distribution stability of the SPI solution. It is found that the SPI particle size can be effectively reduced by high-pressure homogenization, and the minimum size is 330.6 nm. It is suggested that the strong mechanical action in the process of high-pressure homogenization promotes the molecular collision of the SPI system, resulting in a reduction of the SPI particle size. However, when the homogenization pressure is increased to 14 MPa, the molecular collision effect of the SPI system is enhanced due to the increasing degree of homogenization, the system temperature rises, and some SPI thermal aggregation occurs, so that the particle size slightly increases. 

**Figure 1:**
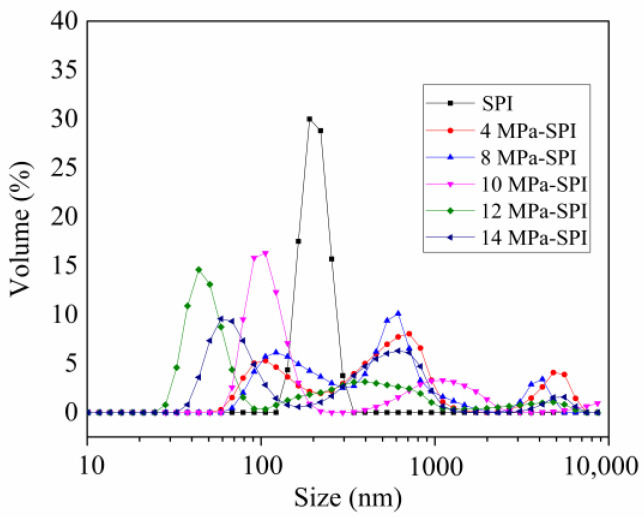
The effect of homogeneous modification on the particle size distribution of soy protein isolate.

Figure 1 shows the effect of high-pressure homogenization on the particle size distribution of the SPI solution. As can be seen from the figure, the untreated samples showed a single peak distribution, while the homogenized samples showed a multi-peak state. The particle size of the sample shifted to the left in the direction of smaller, and the higher the pressure, the more concentrated the particle distribution. However, we found that all samples treated with high-pressure homogenization produced a larger particle size distribution compared to the control group. This may be due to the fact that SPI itself contains a small number of large protein particles, and although they make up a relatively small proportion of the total particle size, they have a significant effect on the particle size distribution of the system. The aggregates generated during the treatment process will also further increase the distribution of large particle sizes. The decrease in the average SPI particle size under the action of homogenization may be mainly related to the change in large particle size and aggregate content. The particle size distribution is consistent with the particle size results shown in Table 1. In addition, Schubert et al. [19] showed that the dispersion of proteins is affected by many factors, such as protein particle size, surface hydrophilicity of protein particles, and so on. The change and size of the distribution range of protein particles can reflect the change in the dispersion index (PDI). In general, the PDI value decreases as the particle size decreases and the distribution range narrows. The uniformity of the particle size distribution of the protein solution can be characterized by the value of PDI, and the smaller the value of PDI, the more uniform the particle size distribution of the protein solution. As shown in Table 1, the PDI values of the homogenized samples are significantly lower than those of the untreated SPI (*p* < 0.05), especially at 12 MPa, where the PDI reaches the lowest value of 0.49, indicating that the particle size distribution uniformity of the homogenized SPI is high. 

### 2.2. Analysis of the SDS-PAGE

Mercaptoethanol breaks disulfide bonds and hydrophobic interactions between SPI subunits, resulting in the dissociation of the subunits [20]. Therefore, SDS-PAGE will show any differences between the treated SPI subunit composition and the control group. According to the study, the electrophoretic map of SPI from top to bottom is as follows [21,22]: The electrophoretic bands with molecular weights of approximately 76 kDa, 72 kDa, and 53 kDa are attributed to the α′ subunits, α subunits, and β subunits of 7S, respectively. The electrophoretic bands with molecular weights of 37~42 kDa and 20 kDa are attributed to the acidic and basic subunits of 11S, respectively.

**Figure 2:**
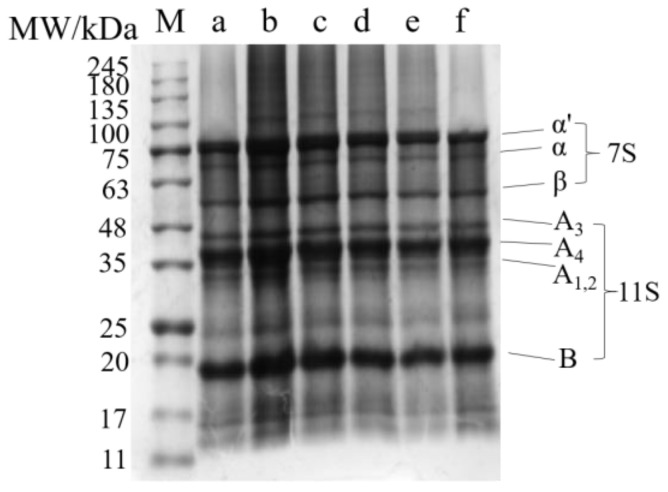
SDS-PAGE spectrum of a homogeneous modified soy protein isolate. M represents the protein marker; a represents the control group SPI; b, c, d, e, and f represent the modified SPI with homogeneous pressures of 4 MPa, 8 MPa, 10 MPa, 12 MPa, and 14 MPa, respectively.

Figure 2 shows the structural composition of SPI subunits under different homogeneous pressures. The findings indicate that the subunits of SPI remain relatively stable across varying homogenization pressures. Although the intensities of the bands were different in the lanes (mainly b, c, and d), this may be caused by a loading mistake. A similar phenomenon was found in the study of Bouaouina et al. [23]: the homogenization pressure of 50–300 MPa did not significantly affect the subunit structure of whey protein, possibly because the time of the homogenization treatment was too short (about 10^−4^ s) and the protein subunits could not be broken into smaller particles. In view of the above analysis, we can speculate that the modification strength of the homogenization treatment is relatively weak, and it will not cause major changes in the subunit structure of SPI. In conclusion, homogenization does not destroy the subunit structure of SPI. However, this modification treatment can effectively cause the development and reconstruction of protein molecules and then change the size of the modified protein molecules.

### 2.3. Analysis of the FT-IR Spectroscopy

It can be seen from Table 2 that, compared with the control group SPI gel, the content of random coil structure of the homogeneously modified SPI gel is significantly decreased, the content of α-helices and β-turns structure is slightly increased, and the content of β-sheet structure is significantly increased, indicating that the homogeneous modification causes the SPI structure to transform into a more β-sheet structure. At the same time, the relative content of the secondary structure of the gel samples changed significantly with the difference in homogeneous pressure treatment. Among all the modified samples, the β-sheet structure content of the 12 MPa treated sample was the highest, while the β-sheet structure content of the SPI in the control group was the lowest. The study by Gao et al. [24] showed that the β-sheet structure in the secondary structure of proteins was significantly positively correlated with the hardness of different gel systems. This conclusion was confirmed in this study, and the hardness value of the samples obtained under 12 MPa homogenization was significantly higher than that of the control group in Table 3 (*p* < 0.05). In addition, Sow et al. [25] showed that increasing the content of random coil structure in gel samples can lead to a decrease in gel hardness. In this study, the relative content of random coil was the highest in the control group, and the relative content of random coil structure was the lowest in the 12 MPa homogeneous modified sample. The results confirmed that the higher the relative content of the random coil, the lower the hardness of the gel sample. It is concluded that the β-sheet structure reflects the order of the secondary structure of soy protein and its gel molecules, and the random coil structure and other structures reflect the disorder of the secondary structure of soy protein and its gel molecules.

It is worth noting that the structural content appears to change with increasing pressure, but this change is not linear. In particular, the β-sheet content peaks at 12 MPa and then decreases to 14 MPa. This could be attributed to the relatively weak transformation from random coil structures to β-sheet structures at this particular pressure. High-pressure homogenization of the sample will improve the hardness of the gel to a certain extent. In particular, the 12 MPa treatment can be used as a theoretical basis for the relationship between structural changes and texture properties of the modified protein, which can effectively regulate the hardness of the gel in actual production. In addition, combined with the study of gel properties, it can be found that the change in secondary structure of soy protein will lead to significant changes in its functional properties, especially the increase in β-sheet content, which is conducive to the improvement of functional properties [7].

### 2.4. Two-Dimensional Fluorescence Spectroscopy Analysis

**Figure 3:**
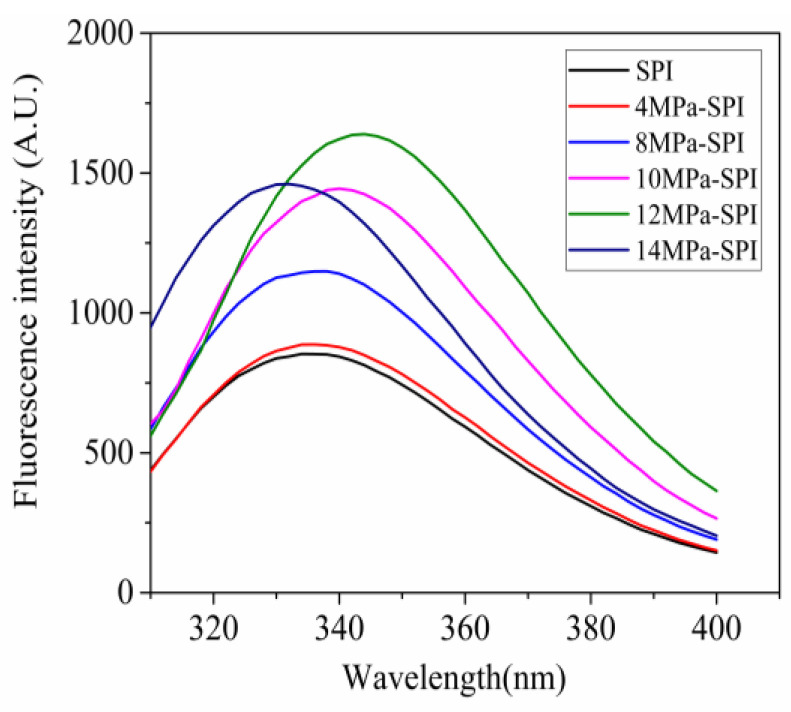
Fluorescence spectra of soy protein isolates under different homogenization pressures.

Figure 3 shows the fluorescence spectral analysis of SPI under different homogeneous pressures. In SPI, phenylalanine, tryptophan, and tyrosine can produce fluorescence, and the largest fluorescence intensity is tryptophan, so the fluorescence of SPI samples is mainly from tryptophan [7]. As shown in the figure, the peak fluorescence λ_max_ of SPI in the control group was 334 nm, while the peak fluorescence λmax of SPI in the homogeneous 4, 8, 10, 12, and 14 MPa treatments were 336, 338, 340, 346, and 332 nm, respectively. The peak of the homogeneous modified SPI shifted to different degrees. When the homogeneous pressure was 12 MPa, the peak redshifted to the largest, and the fluorescence intensity was the largest. The reason for this phenomenon may be that under this homogenization pressure, the protein structure of SPI gradually unfolds, the spatial conformation changes, and more tryptophan residues are exposed to the surface of the protein molecules, i.e., the polarity of the microenvironment of the fluorescence-emitting group increases, and the peptide chain is more extended, resulting in changes in fluorescence shift and intensity. When the homogenization pressure is increased to 14 MPa, the λ_max_ blue shift of the SPI occurs, which may be caused by the subunit reaggregation behavior of proteins in this state. When the hydrophobic groups inside the protein molecules are exposed, λ_max_ is redshifted and increases, but when the protein molecules are aggregated and the structure tends to be tight, the hydrophobic groups are embedded inside the molecule, and λ_max_ is blue-shifted and decreases. It can be confirmed that homogeneous treatment can change the spatial conformation of SPI.

### 2.5. Effect of Different Homogeneous Pressure Treatments on the Water Holding Capacity of Soy Protein Gel

**Figure 4:**
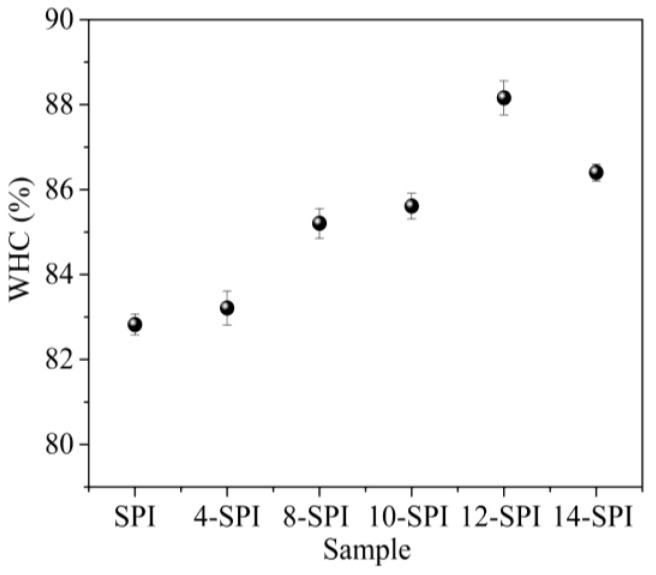
The effect of homogeneous modification on the water-holding capacity of soy protein isolate gel.

Figure 4 shows the water holding capacity (WHC) value of the homogeneously modified gel. The gel WHC reached its maximum after homogenization at 12 MPa, but when the pressure was increased to 14 MPa, the gel WHC decreased. In addition, the WHC values of the homogenized gel were higher than those of the control SPI gel at all pressures (*p* < 0.05). Molina et al. [26] reported similar results, with a significant increase in WHC with increasing pressure in the low pressure range and a decrease in WHC with increasing pressure in the high pressure range. The reason is that the homogeneous pressure causes the molecular structure of SPI to unfold, changing the subunits and aggregation structure of SPI, and the partial exposure of hydrophobic groups enhances the hydrophobic effect, which is beneficial for the formation of gels. The structure becomes more compact, and WHC is good. In addition, when a stronger gel is formed, it can more effectively trap and retain water during centrifugation, resulting in an increase in WHC [27]. However, when the homogeneous pressure is high, SPI forms partially insoluble aggregates, and the particle size and functional properties of the insoluble components are different, so SPI mostly forms irregular aggregates, resulting in uneven gel network structure, and the increase of aggregation and precipitation reduces WHC [28].

### 2.6. Analysis of Texture Properties of Gel Samples

It can be seen from Table 3 that homogeneous modification treatment has a significant effect on the hardness of SPI gel (*p* < 0.05), and the hardness of homogeneously modified SPI gel shows an increasing trend, which may be due to the reduction of particle size of soluble aggregates in SPI by homogenization from 4–14 MPa (compared with the control group). In addition, homogenization may cause partial unfolding and denaturation of SPI, which may enhance its interaction with proteins. With the increase in homogenization pressure, the springiness of SPI gel showed a trend of first increasing and then decreasing, and the springiness of gel treated with 12 MPa was the largest. This is because with the increase in homogenization pressure, SPI denaturation became more complete, which promoted the formation of a dense gel network structure, and both gel hardness and springiness increased. However, when the homogenization pressure increases to a certain extent, protein aggregation occurs and the gel properties show a slight decrease, resulting in the destruction of the dense and ordered network structure of the gel and a significant decrease in its springiness [29]. When the homogeneous pressure is 14 MPa, the hardness of the gel continues to increase, but the springiness decreases. The occurrence of this phenomenon can be explained by the formation of insoluble aggregates caused by the high homogeneous pressure modification [30]. The internal structure of insoluble aggregates produced by homogeneous modification is generally relatively dense, which makes them difficult to break even under strong external stress. The gel springiness is poor at the macroscopic level, and the gel cohesiveness and chewiness are increased, which will show a higher hardness value. The results are consistent with the particle size distribution and SEM results.

### 2.7. Analysis of Rheological Properties of Gel Samples

**Figure 5:**
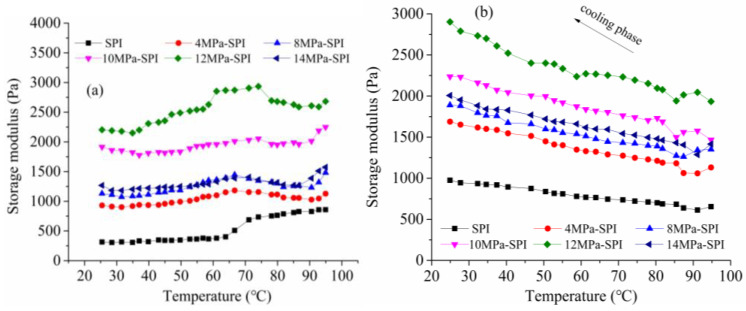
The effect of different homogenization conditions on the rheological properties of soy protein gel temperature sweep. (**a**) shows the heating process; (**b**) shows the cooling process.

In the experiment, the storage modulus (G′) was measured by increasing and decreasing the temperature of the control program to study and analyze the rheological properties of the SPI gel during its formation [31]. Figure 5 is a temperature scan of the influence of different homogeneous pressures on the rheological properties of the SPI gel. As can be seen from Figure 5a, the G′ values of all samples showed a slightly decreasing trend with increasing temperature at the initial stage. The reason for this phenomenon may be that as the temperature increases, the disulfide bonds and hydrogen bonds of the proteins are broken, and the protein molecules are denatured and depolymerized, resulting in a decrease in G′. However, as the temperature continues to rise, G′ rapidly increases. The reason for this phenomenon is that hydrophobic interaction is greatly enhanced at this stage, and a large number of aggregates form and develop into gel network structures, resulting in a sharp increase in G′. Speroni et al. [32] pointed out that the increase in hydrophobic interaction is conducive to the increase in G′. Compared with other gel samples, the G′ of the SPI gel in the control group showed a mutation point at about 70 °C, and that of the modified gel sample appeared at about 60 °C, indicating that homogenization could promote the mutation point of the gel to advance. When the temperature is higher than 90 °C, the G′ value of all samples tends to increase, probably because when the heating temperature exceeds the initial denaturation temperature of the 7S component of SPI (about 72 °C) [33], the denaturation starts to unfold and form part of the fibrous aggregate, and the G′ value starts to accelerate. The denaturation temperature of the 11S component is about 92 °C, and denaturation begins at temperatures above 90 °C [33], resulting in an increase in G′. In addition, Figure 5a shows that the modulus of storage of the SPI gel is significantly changed by homogenization under different pressures during the heating process.

It can be seen from Figure 5b that the storage modulus of all samples increases significantly during the cooling phase. The SPI gel structure is further strengthened and stabilized by hydrogen bonding and van der Waals forces during the cooling phase. Renkema et al. [34] pointed out that the G′ value of SPI shows a strong increase during the cooling phase. The G′ value of the SPI gel after homogeneous modification was higher than that of the control gel sample, especially the Young′s modulus of the SPI gel treated with 12 MPa homogeneous modification, which was significantly higher than that of the control sample, and the rate of increase was the fastest. The results showed that the gel strength of SPI was improved by the homogenization treatment, which was mainly due to the reduction of the aggregation size of soybean protein molecules induced by the homogenization treatment. However, when the homogenization pressure was 14 MPa, the increase in the G′ value of soy protein in the cooling stage slightly decreased, which was mainly due to the inconsistency in the fineness of the gel network structure caused by different homogenization modification treatments. Compared with the fine gel network structure, hydrogen bonding has less effect on the inhibition of protein molecular mobility in the coarse gel network structure [5].

**Figure 6:**
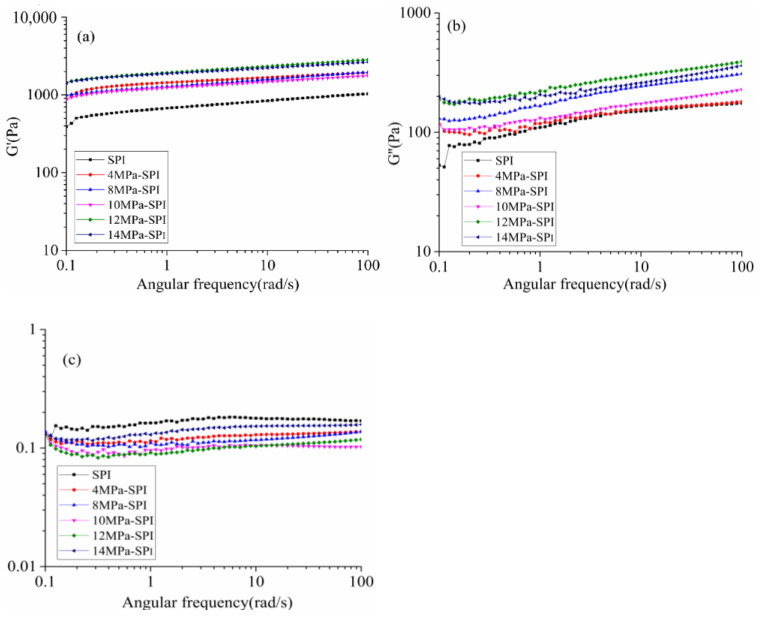
The effect of different homogenization conditions on the rheological properties of soy protein gel frequency sweep. (**a**–**c**) show the variation of G′, G″, and tanδ with the dynamic oscillation frequency, respectively.

Figure 6 shows the frequency scan of the final gel formation, where figures (a–c) show the variation of G′, G″, and tanδ with the dynamic oscillation frequency, respectively. It can be seen from the figure that the scanning patterns of the gel samples are similar in shape, and the G′ and G″ of all the samples increase with the increase of the scanning frequency. The frequency dependence of the G′ and G″ values is consistent with previously observed results for different types of hydrocolloidal soybean protein gels [35,36]. From the numerical value, it can be seen that the gel prepared by SPI treated with 12 MPa homogeneous modification has the highest G′ and G″ values. The G′ value of the homogeneously modified SPI gel is higher than the G″ value, so it can be assumed that all samples have more “solid” properties.

The change in rheology can also be explained by the loss factor (G″/G′ = tanδ). Xiong et al. [37] pointed out that in the formation process of protein gel matrix, tanδ is the relative distribution of “viscosity” to “elasticity”, and the higher the tanδ value, the lower the elasticity of the gel. It can be seen from Figure 6c that the tanδ value of all homogeneous modified gel samples increases with increasing frequency. The results of tanδ < 1 show that the modified gel exhibits solid elasticity under different homogeneous pressures. At 14 MPa homogeneous pressure, the tanδ value is larger than that at other homogeneous pressures, and the tanδ value increases with increasing frequency. The results show that the homogeneous modified gel at 14 MPa has relatively poor bulk elasticity. In addition, the final G′ and G″ values of the SPI gel in the control group were approximately 1031.83 Pa and 175.08 Pa, respectively, corresponding to a tanδ of 0.17. When SPI was modified by different homogeneous pressures to produce cold-induced gels, the corresponding final tanδ values corresponding to 4 Mpa, 8 Mpa, 10 Mpa, 12 Mpa, and 14 Mpa were 0.14, 0.14, 0.11, 0.1, and 0.16, respectively. Our results show that the homogeneous modification can significantly improve the elasticity of the cold-induced gels compared to the control SPI gels. In conclusion, the frequency scanning data further proved that homogeneously modified SPI could improve the gel strength and elasticity of cold-induced Chiba tofu gel.

### 2.8. Intermolecular Force Analysis of Gel Samples

**Figure 7:**
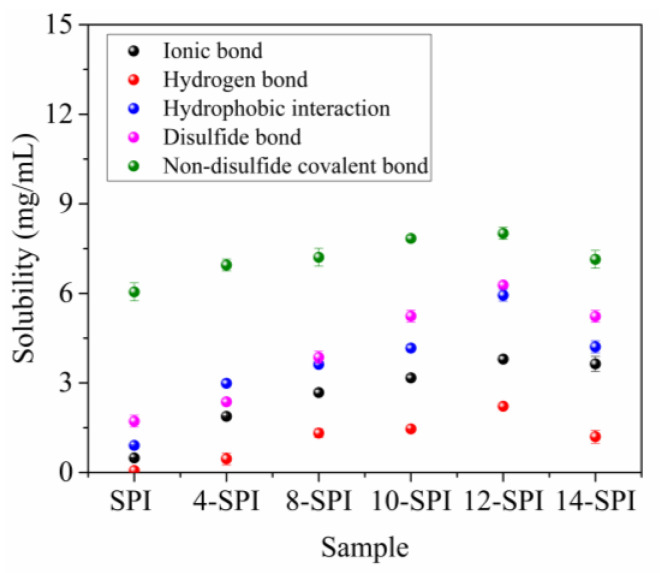
The influence of homogenous modification on the intermolecular force in gel.

Figure 7 shows the relative proportions of non-disulfide covalent bonds, hydrogen bonds, ionic bonds, hydrophobic interactions, and disulfide bonds. Protein gelation is mainly caused by the cross-linking of polypeptide chains, forming a gel structure stabilized by different molecular forces [38]. At the same time, it has been pointed out that hydrophobic associations, disulfide bonds, and hydrogen bonds play an important role in promoting coagulation in protein cross-linking in the gel system [39]. The results show that when the sample is treated with different homogeneous pressures, the numerical order of different types of bonds in the control gel and the modified SPI gel is as follows: Non-disulfide covalent bond > disulfide bond > hydrophobic interaction > ionic bond > hydrogen bond, which is consistent with the results of hydrophobic interaction, electrostatic interaction, and disulfide bond interaction as pointed out by Wang et al. [35].

In addition, with the increase in pressure, the above chemical bond content showed a trend of first increasing and then decreasing. When the pressure was 12 MPa, the content of all chemical bonds reached its maximum value, which may be due to the increase in homogeneous pressure causing partial denaturation of protein molecules, resulting in protein unfolding, exposing hydrophobic residues and internal sulfhydryl groups, and increasing the interaction between molecules. Increases the hydrophobicity of the protein and forms disulfide bonds [40]. Lee et al. [41] showed that the increase in disulfide bonds contributed to the increase in solubility. The study by Yildiz et al. [42] further showed that the disulfide bond content of homogenized and treated samples was still higher than that of the control group. This shows that the collapse-pressure-refolding process can enhance the modification effect and thus effectively change the functional properties of SPI, which is consistent with the textural characteristics of the gel and the WHC results. However, no matter how the structure of SPI is modified, hydrogen bonding is an important interaction force in the protein gel system. The hydrogen bonding values of all homogeneous modified sample gels were higher than those of control SPI gels. Niu et al. [39] reported that the increase in protein solubility in the gel system confirmed the involvement of hydrogen bonding in the gel network. Therefore, disulfide bonds, hydrophobic interactions, and non-disulfide covalent bonds are the main intermolecular forces involved in the formation of homogeneous modified gel samples.

### 2.9. SEM Analysis of the Gel Samples

**Figure 8:**
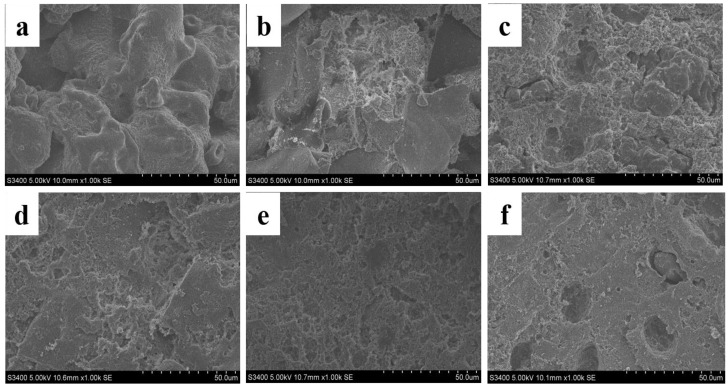
The microstructure of the gel modified by different homogeneous pressures ((**a**) represents the control group SPI gel, (**b**–**f**) represents SPI gels modified with homogeneous pressures of 4 MPa, 8 MPa, 10 MPa, 12 MPa, and 14 MPa, respectively).

As shown in Figure 8, SEM images of SPI cold-induced gel before and after treatment with different homogeneous pressures. As shown in the figure, after magnification of 1000× by SEM, it can be clearly observed that the surface of the gel sample in the control Figure 8a is uneven and has many large and irregular holes. After homogenization at 4 MPa (Figure 8b), the surface of the gel sample was slightly improved but still contained large sheet structures; after homogenization at 8, 10, 12, and 14 MPa, the spatial morphology of the gel samples in Figure 8c–f showed a more regular structure. In particular, the gel sample of Figure 8e treated with 12 MPa homogenization has a more ordered, uniform, and dense structure. This may be because the SPI particle size decreases after homogenization, and the SPI with a small particle size can form a structure with more dense space. In addition, homogenization exposes some active groups, such as sulfhydryl groups and hydrophobic groups, to the surface of the protein molecule or polymer, which promotes the formation of intermolecular disulfide bonds during gel formation and hydrophobic interactions to form a denser and more uniform gel structure.

## 3. Conclusions

The high-pressure homogenization technology to improve the gel performance of SPI is studied in the pressure range closer to the actual production (4–14 MPa), bridging the gap between theoretical research and practical application. The springiness, hardness, cohesiveness, chewiness, and water holding capacity of the modified gel were higher than those of the control group. FT-IR analysis showed that the content of β-sheet structure was significantly increased (*p* < 0.05), the content of α-helix and β-turn structure was slightly increased, and the content of random coil structure was significantly decreased (*p* < 0.05). Molecular force results showed that disulfide bonds, hydrophobic interactions, and non-disulfide bonds are the main intermolecular forces that maintain the structure of the gel network. SEM analysis showed that, compared with the control gel, the modified gel was more uniform and smoother, with smaller pores and a denser structure, especially when the homogenization pressure was 12 MPa. However, in this pressure range, the higher the pressure, the better the effect, but the effect decreases when the homogenization pressure is 14 MPa, possibly because the pressure is too high, resulting in insoluble aggregates. This research is limited to one SPI feedstock and may be extended to dozens of feedstocks in the future to further determine the general applicability of high-pressure homogenization technology. The sustainability of SPI functional properties improved by high-pressure homogenization technology, as well as the taste, mechanism, and thixotropic behavior of gel products, also need to be further investigated, which will provide a comprehensive discussion and practical theoretical basis for effectively improving high-pressure homogenization technology to promote the functional properties of food.

## 4. Materials and Methods

### 4.1. Materials

SPI (protein content, 98%) was provided by Yuwang Ecological Food Industry Co., Ltd. (Dezhou, Shandong, China). Glutamine transaminase (TG; 100 units (U)/mg), bovine serum albumin (BSA), and protein markers were purchased from Solarbio and Technology Co., Ltd. (Beijing, China). Other reagents were purchased from Terry Experimental Supplies Co., Ltd. (Harbin, Heilongjiang, China) and were at least analytical grade.

### 4.2. Preparation of SPI Modified by High-Pressure Homogenization Technology

The SPI raw material was dissolved in deionized water to prepare a 7.5% (*w/v*) protein solution, which was stirred at room temperature for 2 h to allow complete dissolution. The solution was divided into 6 groups; 1 group was not treated as a control group, and the other 5 groups were homogenized under high pressure of 4, 8, 10, 12, and 14 MPa, respectively. High-pressure homogenizers (SPCH-10, Axa United Technologies Ltd., Shanghai, China) are usually equipped with temperature control systems (e.g., jacket temperature control) to ensure that the sample is homogenized within the appropriate temperature range. The obtained solution was frozen overnight at −18 °C and then lyophilized in a freeze dryer to obtain the final homogeneous modified powder sample. The control samples and the samples obtained after different 4, 8, 10, 12, and 14 MPa treatments were designated as SPI, 4 MPa-SPI, 8 MPa-SPI, 10 MPa-SPI, 12 MPa-SPI, and 14 MPa-SPI, respectively.

### 4.3. Preparation of Cold-Induced Gel (Chiba tofu)

The sample preparation of Chiba tofu was divided into four steps based on the methodology proposed by Xu et al. [8]. The first step is the preparation of the slurry, as follows: SPI (15 g), potato starch (5 g), and distilled water (80 mL) were mixed in a Braun mixer at 1000 rpm for approximately 3 min. TG (20 U/g) was added at a slow speed, and the mixture was stirred at a speed of 3000 rpm for about 2 min until uniform. Furthermore, NaCl (0.2 g), monosodium glutamate (0.1 g), and soybean oil (1 mL) were added at a slow speed, and the slurry was stirred at a speed of 9000 rpm for 5 min to make the slurry uniform and free of small bubbles. An ice bath was used to keep the temperature of the whole process below 12 °C. During the aging process, the slurry was carefully poured into a tray (26 × 36 cm) with a thickness of 4 cm. The slurry surface was covered with plastic film and placed in the refrigerator (about 4 °C) for 10 h. The aging sample is placed in a water bath at a constant temperature of 50 °C for 45 min. Finally, the sample tray is immersed in a water bath (80–85 °C) for 40 min so that the center temperature of the product reaches 75 °C or more. Allow the sample to cool to room temperature before slicing.

### 4.4. Determination of Particle Size and Distribution

The lyophilized control and homogenized SPI powder were dissolved in deionized water to form a 1 mg/mL solution and stirred at room temperature. To allow for complete dissolution, the solution was left overnight at 4 °C. A dynamic light scattering (DLS) instrument (Malvern, Panalytical Ltd., Shanghai, China) was used to measure particle size, which was expressed as volume average diameter D_[4,3]_.

### 4.5. Distribution and Determination of Sodium Dodecyl Sulfate Polyacrylamide Gel Electrophoresis (SDS-PAGE)

The SPI solutions (2 mg/mL, 1 mL) were prepared, and 40 µL were added to 2× SDS-PAGE sample buffer (containing β-mercaptoethanol) in the UP tube (1 mL) at a ratio of 1:1 (*v*/*v*) and thoroughly mixed. The mixed samples were heated in boiling water (>90 °C) for 5 min. After cooling, the sample loading volume of 20 µL was added with a pipette. SDS-PAGE analysis was performed using an SDS-Tris-glycine buffer system with 15% separating gels and 5% stacking gels. Electrophoresis was performed at 80 V in the stacking gel and was increased to 120 V when the samples entered the separating gel until the tracking dye reached the bottom of the gel using a horizontal electrophoresis system (Deyuan Co., Ltd., Harbin, Heilongjiang, China). At the end of electrophoresis, the samples were stained with 0.25% Coomassie Bright Blue R-250 (for 0.5 h) and destained (glacial acetic acid/methanol/water = 1:1:8 (*v*/*v*/*v*)) (destaining for 30 min, twice, and then overnight) [7]. Protein bands in the samples were scanned using a Gel Doc EZ imager (Bio-Rad Laboratories, Shanghai, China) gel imaging system, assuming a linear response and using the imager’s peak area algorithm.

### 4.6. Determination of Fourier Transform Infrared Spectroscopy (FT-IR)

Untreated SPI (3 mg) and high-pressure homogenized SPI (3 mg) were weighed and mixed with KBr powder in a ratio of 1:100 (*w/w*). The mixture was pressed through a tablet press. The sample was scanned using a Nicolet iS10 FT-IR spectrometer (Thermo Fisher Scientific, Waltham, MA, USA) with a resolution of 4 cm^−1^, a scan band of 4000–400 cm^−1^, and scan times of 16. The scans from 1600 to 1700 cm^−1^ are divided into the amide I bands. The “peak fitting″ method was used to quantitatively analyze the secondary structural components of the samples using Peak Fit v4.12 derivative function software (Origin Lab Corp., Waltham, MA, USA). The four structures containing α-helix (1650–1660 cm^−1^), β-sheet (1618–1640 and 1670–1690 cm^−1^), β-turn (1660–1700 cm^−1^), and random coil (near 1645 cm^−1^) were obtained [43].

### 4.7. Determination of the Fluorescence Spectrum

An F-4500 fluorescence spectrophotometer (Hitachi Corporation, Tokyo, Japan) was used to measure the fluorescence spectrum of SPI. The sample (0.2 mg/mL) was prepared with 10 mL of 0.01 mol/L sodium phosphate buffer (pH 7.0). The experimental parameters were set as follows: excitation wavelength was 280 nm, emission spectrum was 300–400 nm, data interval was 2 nm, scan speed was 2000 nm/min [35], and both excitation and emission gaps were 10 nm.

### 4.8. Determination of the Water-Holding Capacity of Gels

A cold-induced gel sample (5 g) was placed in a 50-mililiter polypropylene conical tube, weighed, and recorded as W_1_. The next step was centrifugation. The parameters were set to 3000× *g*, 4 °C, 15 min. The water is removed, and the centrifuge tube containing the sample is weighed and recorded as W_2_. The weight of water removed from the gel sample is recorded as W_r_ (W_r_ = W_1_ − W_2_). In the experiment, the total weight of water in the gel sample was measured by heating the gel sample in an oven at 105 °C for 2 h, which was recorded as W_0_. The formula for WHC is as follows [44]:

$$WHC=\frac{{W_{0}-W}_{r}}{W_{0}}\times 100$$

### 4.9. Determination of TPA (Texture Profile Analysis) of Gels

According to the test method of Zhao et al. [45], the hardness, springiness, chewiness, and cohesiveness of the cylindrical specimen after 50% deformation (trigger force of 5 g) were determined using a P/36R probe (diameter of 36 mm). The test parameters were set as follows: initial speed of 1.5 mm/s, test speed of 2.0 mm/s, and post-test speed of 2.0 mm/s (Model TA-XT2 Texture Analyzer, Stable Micro Systems Ltd., Godalming, UK). The analysis was repeated three times for each specimen. The texture parameters were calculated using the instrument’s software (Nexygen Plus analysis software 40/0783).

### 4.10. Determination of Rheological Properties of Gel Samples

The rheology was determined by the method of Li et al. [10]. In this study, temperature scanning and frequency scanning were performed. Samples were analyzed in temperature scan mode using a DHR-3 rheometer with parallel plates (TA Instruments, Waters Technology Corporation, Milford, Massachusetts, USA). After mixing the modified protein solution with the TG using a scroll oscillator, the sample was injected between the plates. When the plates were filled with the solution, the excess sample was removed, silicone oil was applied, and a protective cap was added. The maximum strain was 0.01, and the oscillation frequency was kept at 1 Hz. The sample was heated from 25 °C to 95 °C at 2 °C/min, held at 95 °C for 20 min, and then cooled to 25 °C at 2°C/min. The storage modulus (G′) was recorded. At the end of the temperature scanning, the temperature was kept at 25 °C, the deformation was fixed at 0.01, the scanning frequency was from 0.1 to 100 rad/s, and the parameters such as G′ and G″ were measured.

### 4.11. Determination of Gel Intermolecular Force

According to the method of Yang et al. [46], the gel sample (2 g) was minced and mixed with 10 mL of S1 solution (0.6 M NaCl, adjusted to pH 7.0 with 0.01 M NaOH and 0.01 M HCl), then homogenized for 2 min using an IKA homogenizer set at 8 (approximately 18,000 rpm). The mixture was stirred at 4 °C for 1 h and then centrifuged (15,000× *g*, 4 °C) for 20 min. The supernatant was stored at 4 °C. Solution S2 (10 mL, 0.6 M NaCl + 1.5 M urea) was added to the precipitate obtained by centrifugation, and the above steps were repeated. Solution S3 (10 mL, 0.6 M NaCl + 8 M urea) was added to the precipitate obtained by centrifugation, and the above steps were repeated. Solution S4 (10 mL, 0.6 M NaCl + 8 M urea + 0.5 M β-mercaptoethanol) was added to the precipitate obtained by centrifugation, and the above steps were repeated. Finally, NaOH solution (2 mL, 1 mol/L) was added to the precipitate obtained and stored at 4 °C (S5). Each supernatant was mixed with an equal volume of 20% trichloroacetic acid and centrifuged at 5500× *g* for 15 min at 4 °C. Each precipitate was dissolved in 2 mL of 1 M NaOH and stored at 4 °C. Protein content was determined by the Biuret method using BSA as a standard [46], assuming a purity of 100%, and results were expressed as BSA equivalents. Solubilities measured in solvents S1, S2, S3, S4, and S5 represent proteins solubilized by disruption of ionic, hydrogen, hydrophobic, disulfide, and non-disulfide bonds, respectively.

### 4.12. Determination of Microstructure of Gel Samples

Following the method of Lin et al. [47], the morphology and microstructure of the gel were observed by SEM (SU6600, Hitachi High Technologies Corp., Tokyo, Japan). The gel was cut into small 2 × 5 mm^2^ pieces with a sharp, double-sided blade and fixed in 0.1 M sodium phosphate buffer (pH 6.8) containing 2.5 M glutaraldehyde for 4 h in a refrigerator at 4 °C overnight. After a series of rinses, the gel sample is lyophilized. A metal film was then applied to the gel using an ion-sputtering coater (E-1010, HITACHI). The images were then observed at an accelerated voltage of 5.0 kV, and the XT microscope control version 2.5.0.3 software was used to collect the images at 1000× magnification.

### 4.13. Statistical Analysis

Measurements were repeated three times for each experiment, and the data obtained were expressed as mean ± standard deviation (SD). SPSS version 20 software (SPSS, Chicago, IL, USA) was used for analysis of variance (ANOVA) differences, followed by Duncan’s multiple comparison test, and *p* < 0.05 was considered significant. Origin Pro 2016 64-bit software (Origin Lab Corp., Northampton, MA, USA) was used for the drawings in the experiment.

## Figures and Tables

**Table 1:** The influence of different homogenization pressures on the particle size of soy protein isolate.

Sample	D_[4,3] _ (nm)	PDI
SPI	2311.33 ± 40.00 ^a^	0.81 ± 0.04 ^a^
4 MPa-SPI	378.34 ± 60.00 ^b^	0.73 ± 0.02 ^b^
8 MPa-SPI	367.20 ± 30.00 ^b^	0.71 ± 0.03 ^b^
10 MPa-SPI	364.63 ± 20.00 ^b^	0.54 ± 0.01 ^c^
12 MPa-SPI	330.60 ± 10.00 ^b^	0.49 ± 0.02 ^d^
14 MPa-SPI	335.23 ± 10.00 ^b^	0.50 ± 0.03 ^cd^

Note: SPI stands for untreated control group. Moreover, 4 MPa-SPI, 8 MPa-SPI, 10 MPa-SPI, 12 MPa-SPI, and 14 MPa-SPI represent the modified SPI with homogeneous pressures of 4 MPa, 8 MPa, 10 MPa, 12 MPa, and 14 MPa, respectively. Different lowercase letters in the same column indicate significant differences (*p* < 0.05). D_[4,3] _ represents the average particle size of the protein, and PDI represents the protein dispersion index.

**Table 2:** The contents of the secondary structures of gel products.

Sample	α-Helix (%)	β-Sheets (%)	β-Turns (%)	Random Coil (%)
SPI	16.00 ± 0.10 ^c^	50.10 ± 1.10 ^b^	14.10 ± 0.10 ^d^	19.80 ± 1.00 ^a^
4 MPa-SPI	16.84 ± 0.03 ^a^	50.80 ± 1.00 ^b^	15.54 ± 0.02 ^a^	16.83 ± 0.03 ^b^
8 MPa-SPI	16.83 ± 0.04 ^a^	51.00 ± 1.00 ^ab^	15.36 ± 0.01 ^b^	16.81 ± 0.02 ^b^
10 MPa-SPI	16.82 ± 0.10 ^a^	51.06 ± 1.42 ^ab^	15.40 ± 0.01 ^b^	16.72 ± 1.00 ^b^
12 MPa-SPI	16.91 ± 0.10 ^a^	53.12 ± 1.00 ^a^	14.93 ± 0.09 ^c^	15.04 ± 0.04 ^c^
14 MPa-SPI	16.35 ± 0.02 ^b^	51.43 ± 1.20 ^ab^	15.45 ± 0.03 ^ab^	16.77 ± 1.00 ^b^

Note: SPI stands for untreated control group. Moreover, 4 MPa-SPI, 8 MPa-SPI, 10 MPa-SPI, 12 MPa-SPI, and 14 MPa-SPI represent the modified SPI with homogeneous pressures of 8 MPa, 10 MPa, 12 MPa, and 14 MPa, respectively. Different lowercase letters in the same column indicate significant differences (*p* < 0.05).

**Table 3:** The effect of homogeneity on the textural properties of the SPI gel.

Different Pressure/MPa	Hardness/Pa	Springiness	Cohesiveness	Chewiness/Pa
SPI	2.76 ± 0.07 ^e^	0.75 ± 0.04 ^c^	0.91 ± 0.01 ^c^	1.88 ± 0.14 ^e^
4 MPa-SPI	7.70 ± 0.10 ^d^	0.81 ± 0.02 ^b^	0.93 ± 0.04 ^bc^	5.80 ± 0.10 ^d^
8 MPa-SPI	8.41 ± 0.05 ^c^	0.83 ± 0.04 ^b^	0.9 ± 0.01 ^abc^	6.63 ± 0.20 ^c^
10 MPa-SPI	8.60 ± 0.10 ^c^	0.84 ± 0.03 ^b^	0.97 ± 0.01 ^ab^	7.01 ± 0.10 ^b^
12 MPa-SPI	9.10 ± 0.20 ^b^	0.90 ± 0.02 ^a^	0.97 ± 0.03 ^ab^	7.94 ± 0.10 ^a^
14 MPa-SPI	10.90 ± 0.10 ^a^	0.76 ± 0.01 ^c^	0.98 ± 0.02 ^a^	8.01 ± 0.04 ^a^

Note: SPI stands for untreated control group. Moreover, 4 MPa-SPI, 8 MPa-SPI, 10 MPa-SPI, 12 MPa-SPI, and 14 MPa-SPI represent the modified SPI with homogeneous pressures of 8 MPa, 10 MPa, 12 MPa, and 14 MPa, respectively. Different lowercase letters in the same column indicate significant differences (*p* < 0.05).

## Data Availability

Data are contained within the article.
